# Supplementary material for: MetaRibo-Seq measures translation in microbiomes
Source: Nat Commun. 2020 Jun 29;11:3268. doi: 10.1038/s41467-020-17081-z (PMC7324362; doi:10.1038/s41467-020-17081-z)
Supplement: Supplementary file 10 — Supplementary Data 7 [file 41467_2020_17081_MOESM10_ESM.zip › File2/Confidence_High_Taxonomy/199767_out.krona.html]

Javascript must be enabled to view this page.

members
magnitude
magnitudeUnassigned
count
unassigned
taxon
rank

199767\_out

96

superkingdom
96
2

96
phylum
1239

class
96
186801

8
186802

SRS043411\_contig\_number\_contig-100\_762.109674SRS044535\_contig\_number\_23907SRS049712\_contig\_number\_25123SRS054905\_contig\_number\_25429SRS077024\_contig\_number\_12350SRS077730\_contig\_number\_16203SRS143780\_contig\_number\_31995SRS145497\_contig\_number\_contig-100\_322.126295
order
96


SRS048060\_contig\_number\_contig-100\_678.84240SRS053649\_contig\_number\_contig-100\_777.142006SRS144362\_contig\_number\_18479SRS147425\_contig\_number\_contig-100\_1211.83241SRS893230\_contig\_number\_10145
family
5
186803
75

572511
genus
2

33039
species

SRS893231\_contig\_number\_contig-100\_37.49645
1

2292969

SRS097958\_contig\_number\_22370
1
species

genus
1
1432051

2292348

SRS017916\_contig\_number\_16983
1
species

30

SRS011084\_contig\_number\_contig-100\_461.214201SRS021948\_contig\_number\_33958SRS1055022\_contig\_number\_21652
genus
3
841

3

SRS011134\_contig\_number\_contig-100\_124.311657SRS050925\_contig\_number\_38065SRS143466\_contig\_number\_15105
species
301302

512314
species

SRS098717\_contig\_number\_21482SRS893373\_contig\_number\_24189
2


SRS077335\_contig\_number\_8815
1
species
2049040

species

SRS011239\_contig\_number\_contig-100\_275.173101SRS013951\_contig\_number\_26798SRS014287\_contig\_number\_11200SRS014412\_contig\_number\_9187SRS014459\_contig\_number\_40139SRS014923\_contig\_number\_contig-100\_336.246813SRS019161\_contig\_number\_40851SRS019445\_contig\_number\_contig-100\_318.205882SRS022137\_contig\_number\_22285SRS047044\_contig\_number\_contig-100\_1282.102606SRS052027\_contig\_number\_18679SRS053335\_contig\_number\_contig-100\_487.114658SRS063040\_contig\_number\_17661SRS098061\_contig\_number\_contig-100\_199.93440SRS1041144\_contig\_number\_313SRS104693\_contig\_number\_contig-100\_126.96748SRS146812\_contig\_number\_contig-100\_257.321649SRS149075\_contig\_number\_contig-100\_415.119644SRS893270\_contig\_number\_17025
19
166486

species

SRS016989\_contig\_number\_17306
1
301301

360807
species
1

SRS097920\_contig\_number\_11381

1898203

SRS893285\_contig\_number\_1876
1
species

39491

SRS013687\_contig\_number\_34946SRS015065\_contig\_number\_33457SRS015264\_contig\_number\_23014SRS015431\_contig\_number\_68875SRS016018\_contig\_number\_13652SRS016095\_contig\_number\_31472SRS016335\_contig\_number\_39417SRS017307\_contig\_number\_contig-100\_562.182102SRS018836\_contig\_number\_contig-100\_265.166948SRS019496\_contig\_number\_18064SRS022609\_contig\_number\_48403SRS024492\_contig\_number\_20281SRS042284\_contig\_number\_contig-100\_576.52037SRS049446\_contig\_number\_11742SRS055982\_contig\_number\_18361SRS056259\_contig\_number\_16806SRS058723\_contig\_number\_contig-100\_312.63547SRS065176\_contig\_number\_16493SRS075773\_contig\_number\_contig-100\_90.238809SRS075878\_contig\_number\_24389SRS098073\_contig\_number\_17739SRS104084\_contig\_number\_13369SRS1041037\_contig\_number\_10769SRS104975\_contig\_number\_contig-100\_228.135340SRS1055067\_contig\_number\_14733SRS142542\_contig\_number\_25367SRS142980\_contig\_number\_5103SRS144714\_contig\_number\_25516SRS147139\_contig\_number\_contig-100\_52.248401SRS148319\_contig\_number\_contig-100\_346.101161SRS148784\_contig\_number\_contig-100\_733.168821SRS148817\_contig\_number\_14033SRS149325\_contig\_number\_contig-100\_253.102988SRS149784\_contig\_number\_contig-100\_762.167364SRS893342\_contig\_number\_contig-100\_1016.71477
35
species

species
1

SRS024009\_contig\_number\_7609
2109691

216572
family
1

1
genus
459786

species
1

SRS051031\_contig\_number\_12106
876091

family
3
186806

1730
genus
3

species
2

SRS077641\_contig\_number\_contig-100\_153.136564SRS147346\_contig\_number\_22623
142586

165185
species
1

SRS053214\_contig\_number\_18237

1897042
species
3

SRS017103\_contig\_number\_40663SRS143070\_contig\_number\_12399SRS149181\_contig\_number\_22081

family
6
31979

1485
6
genus

2

SRS015960\_contig\_number\_contig-100\_388.140668SRS142890\_contig\_number\_33415
species
59620

2293014

SRS104485\_contig\_number\_3096SRS104636\_contig\_number\_16033
2
species

1262845
1

SRS104311\_contig\_number\_1542
species

species
1

SRS047014\_contig\_number\_37283
1262777
